# Adaptation of Temperature Profiles in CO_2_ Methanation Reactors by an Appropriate Selection of Catalyst and Dilution Agent

**DOI:** 10.1021/acs.iecr.5c03331

**Published:** 2026-01-21

**Authors:** Matteo Percivale, Mauro Andrea Pappagallo, Emanuele Moioli, Gabriella Garbarino

**Affiliations:** † Dipartimento di Ingegneria Civile, Chimica e Ambientale, Università degli Studi di Genova, Via all’Opera Pia 15, 16145 Genova, Italy; ‡ Center for Energy and Environmental Science, 28498 Paul Scherrer Institute, Forschungsstrasse 111, 5232 Villigen, Switzerland; § Dipartimento di Chimica, 18981 Materiali e Ingegneria Chimica ‘Giulio Natta’, Politecnico di Milano, Piazza Leonardo da Vinci 32, 20133 Milano, Italy; ∥ INSTM, UdR Genova, Via Dodecaneso 31, 16146 Genoa, Italy

## Abstract

The control of temperature in CO_2_ methanation reactors is a challenging task due to the high exothermicity of the reaction and the high reaction rate observed when feeding pure reactants. This study analyses a new concept for moderating the reaction exothermicity by controlled dilution of a Ni-based catalyst using materials with different thermal conductivities. The simple decrease in the concentration of the catalyst active phase is not sufficient to control the temperature in the reactor because of the parametric sensitivity of the reaction, which means that a certain threshold of active phase exists, above which the reaction becomes so fast to cause the formation of a pronounced reaction hotspot and below which the reaction rate is too low to achieve high conversion. Therefore, the range of catalyst active phase concentrations that enables the reactor to remain active while maintaining a temperature below 550 °C using an externally cooled reactor is too narrow for practical applications. To achieve reasonable temperature control and a sufficient CO_2_ conversion, the catalyst could be mixed with an inert solid material that can enhance the axial heat dispersion, so as to induce a good distribution of the heat release over the axial coordinate. This would reduce the localized load to the cooling system and spread it over a larger surface. The conductivity of different standard inert materials was evaluated, showing that Al_2_O_3_ and ZnO are the best options for application in CO_2_ methanation. It was then observed that when the reaction rate decreases due to the approach to thermodynamic equilibrium, the axial dispersion of heat should be reduced to enhance the reactor performance, thanks to a fast temperature reduction. This results in a clear trade-off relationship between the reactor length, to achieve a given conversion, and the hotspot temperature. To break this trade-off, it would be necessary to modify the catalyst dilution characteristic over the axial coordinate, allowing for at least two different catalyst dilution zones.

## Graphical abstract

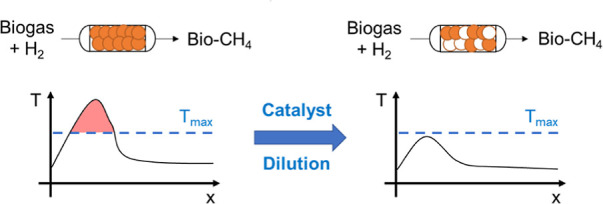

## Introduction

In recent decades, growing concerns about climate change have highlighted the need for an energy transition from fossil fuels to renewable resources. In this context, power-to-gas (PtG) technologies are highly relevant as they allow the production of storable energy carriers (e.g., methane, methanol, or ammonia) using renewable energy.^1,2^ One possibility is represented by the conversion of green H_2_ and CO_2_ into e-methane via the CO_2_ methanation (Meth) reaction (eq 1).

1 $${CO}_{2\left(g\right)}+{4H}_{2\left(g\right)}⇌{CH}_{4\left(g\right)}+{2H}_{2}O_{\left(g\right)},{,\Delta H}_{Meth}^{0}=-165kJ/mol$$

H_2_ can be produced from renewable energy using well-established technologies such as alkaline or proton exchange membrane electrolysis;^3,4^ however, the low density, low boiling point, and high reactivity of H_2_ make it challenging to store^5^ and to be directly used in common applications. On the other hand, there is a well-developed and capillary worldwide distributed infrastructure for storing and transporting methane, both in compressed and liquefied forms.^6^ Therefore, the e-methane, produced by the CO_2_ methanation process, can be injected directly into the natural gas grid once it meets the purity requirements.^7^

The design of an economically feasible process demands a relatively inexpensive source of CO_2_. In this context, the use of biogas as a feedstock for the CO_2_ methanation reactor significantly reduces the operating costs, especially when compared to other carbon-based technologies such as direct air capture.^8,9^ In general, the biogas mixture consists of 50–70% methane, 30–50% CO_2_, and several impurities that vary depending on the source of the biogas. H_2_S, S-based organic compounds, moisture, and other volatile organic compounds may be present, so that biogas purification steps are necessary to avoid compromising the catalytic performance. However, it is easy to find detailed studies in the literature on biogas pretreatment to remove impurities.^10–13^ The methanation reaction can be carried out with pure CO_2_, by accounting for a previous CO_2_ separation from the biogas stream, or directly on the biogas mixture.^14,15^ In the present work, the direct biogas methanation process is evaluated. This allows one to obtain a product stream with potentially 100% methane by converting CO_2_ in the methanation reactor. In this way, methane acts as a heat carrier and significantly reduces the hotspot temperature without having any remarkable disadvantage in terms of the CO_2_ conversion. According to Dannesboe et al.,^16^ CO_2_ conversion decreases by ∼1% when CO_2_ is diluted by 1.75 times CH_4_.

As CO_2_ methanation is a highly exothermic reaction, the formation of a temperature hotspot is almost inevitable in fixed-bed reactors. Therefore, it is necessary to limit the hotspot temperature to prevent catalyst deactivation and performance decrease.^17,18^ For this reason, the maximization of reactor cooling and the specific heat management have been studied in depth in recent years, with the development of large surface area reactors,^8^ vessels equipped with monoliths,^19^ and highly conductive structures.^20^ Additionally, extensive work was performed on the description of the influence of different types of catalyst dilution strategies. Several works of the group of Sundmacher^21–23^ investigated the influence of catalyst particle design on overall reactor performance for flexible and dynamic operations. In particular, the “core–shell” particles with an active core surrounded by an inert and low-permeability shell offer superior thermal stability and dynamic flexibility. This leads to reducing the risk of the formation of an excessive temperature hotspot and minimizing the complexity of the system. Another possibility is represented by the dilution of the catalytic bed to improve the load flexibility, removing the limitations in dynamic conditions.^24–26^ In particular, Fache et al.^24^ and Fache and Marias^25^ demonstrated that tailored dilution or proper catalyst distributions can enhance steady-state efficiency and stability of the reactor. Furthermore, Fischer and Freund^26^ showed that combining these methods with reactor design optimization reduces hotspot formation and enables flexible operation. Therefore, the catalyst dilution influences not only heat transfer but also improves reactor controllability.

Various strategies have been proposed to overcome hotspot formation, including the use of conductive supports, inert diluents, or structured reactors, to enhance heat removal or redistribute heat generation within the catalytic bed. Previous studies have demonstrated that the thermal properties of supports and beds affect hotspot formation. For instance, Petersen et al.^27^ showed that tailored SiO_2_/SiC supports can control surface temperature and selectivity in Ru-based methanation systems. Kulkarni et al.^28^ reviewed the role of SiC as an inert filler and support, highlighting its ability to suppress local hotspots and enhance heat transfer in CO_2_ and CO methanation systems. Furthermore, Jeong et al.^29^ investigated the use of multilayer and dilution strategies as design tools to mitigate hotspots and enhance operational flexibility. Therefore, the present work provides a systematic modeling analysis of axial heat dispersion and catalyst dilution, mapping conversion and hotspot trade-offs across a wide range of materials and proposing a two-layer bed configuration as a practical design compromise. In this way, it is possible to better control the temperature profile, resulting in an improved system performance. This can be carried out by finely tuning the properties of the catalytic packed bed and assessing the heat release, the reactor cooling, and the catalyst composition and dilution. This strategy appears, to the best of our knowledge, to be still unexplored in the literature, and therefore, a thorough modeling study of the effect of the dilution of a Ni-based catalyst with several conductive materials is presented here. In particular, the effects of reducing the quantity of catalyst and diluting it with inert compounds (showing different thermal conductivities) were investigated. The study was performed by simulating several diluting materials, but the results are generalized in terms of the conductivity of the materials so that they can be easily generalized to any kind of diluting agent.

## Methodology

In the present work, a tubular reactor externally cooled with pressurized boiling water was considered. The reactor model has been developed to investigate an industrial system for the direct biogas methanation (CO_2_ + CH_4_), in line with the one proposed by Topsøe in the paper by Dannesboe et al.^30^ The hydrogen converted in the reactor must be produced from renewable and sustainable energy sources (e.g., via renewable-powered water electrolysis). The reactor modeling was performed using Matlab 2023a; reactor characteristics and used equations are detailed in the following paragraphs.

### Methanation Reactor

As mentioned before, the chosen tubular reactor (2.4 m length) operates at 280 °C and 8 bar and is externally cooled by pressurized (65 bar) boiling water at 280 °C (*T*_e_).^30^ The feed consists of a CO_2_/CH_4_/H_2_ ratio of 1:1.5:4, with a total flow rate of 5 N m^3^/h (40 MW of methane produced, based on the HHV). The system is operated with a conventional supported Ni-based catalyst (58 wt %). In the present model, only one pipe of the reactor was considered, for the sake of simplicity. However, as the GHSV, tube characteristic dimensions, and operating conditions remain the same, the scalability of the reactor is only a matter of increasing the number of tubes, and all tubes would exhibit the same profiles. The reactor design and operating parameters of the plant are summarized in Table 1. The model was adapted from the multitubular methanation reactor, based on the model of Schlereth et al.,^31^ by introducing the heat axial dispersion coefficient in the thermal balance, as done by Bremer et al.^18^ According to the same study, the mass axial dispersion term is not needed for an accurate description of the system. The axial parameter allows the description of the heat axial distribution due to the gas (reactants and products flow) and solid (catalytic bed) thermal conductivity. In the present work, it was assumed to be equal to the effective thermal conductivity of the bed since this accounts for the thermal conductivities of both the solid and gas phases (eq 11). Furthermore, according to the literature,^31^ the difference between one-dimensional and two-dimensional pseudohomogeneous reactor models is minor. The 1D model slightly underestimates the peak temperature because it represents a radially averaged value, whereas the 2D models resolve radial gradients. Schlereth and Hinrichsen^31^ reported that the maximum temperature predicted by the 1D model differs by less than 40 °C from the 2D results, corresponding to a relative deviation of less than 7% with respect to the hotspot temperature of approximately 600 °C. Therefore, the 1D model used in this study can accurately represent the behavior of the reactor while avoiding the significant increase in computational complexity associated with 2D simulations.

**Table 1:** Reactor Specifications and Operating Parameters

parameters		units
*P*	8	bar
*T*	280	°C
*T*_e_	280	°C
GHSV	1500	h^–1^
*Q*_tot_	5	Nm^3^/h
*L*	2.4	m
*D*	0.042	m
*d*_P_	0.005	m
S	0.002	m
ε_pb_	0.3	-
Ψ	0.3	-

In this investigation, we focused on understanding the effect of variations in catalyst density and thermal conductivity on the hotspot temperature and the CO_2_ conversion profile. As CO_2_ methanation is 100% selective for methane at low temperatures (as shown in Figure S1a), conversion and temperature are interdependent.^32^ Additionally, it was considered to dilute the catalyst with inert materials characterized by different thermal conductivities. Therefore, the reactor design analysis was performed considering the following design parameters: the dimensions of the reactor, the activity of the catalyst (in terms of the concentration of active phase), the amount of catalyst dilution, and the characteristics of the inert used for catalyst dilution. The presence of inert materials for catalyst dilution in essence changes the heat transfer properties of the catalytic bed as well as its density. The reactor performance was evaluated in terms of space-time yield, considering a maximum allowed temperature of 550 °C and a minimum methane yield of 97%. A pseudohomogeneous reactor model was used, as the influence of heat and mass transfer limitations was assessed through the use of the effectiveness factor η (eq 4), which was calculated based on literature correlations.^33,34^

Thus, the molar (eq 2) and heat (eq 3) values are developed as follows:

2 $$Molar\;balance:\;\frac{{dF}_{i}}{dz}=\rho_{catalyst}\left(1-\varepsilon_{pb}\right)A_{sez}\sum_{j}\nu_{ij}\;f\;{\eta \;r}_{j}$$

3 $$Heat\;balance:\;\lambda_{P}\frac{\partial^{2}T}{{\partial z}^{2}}=\rho_{gas}C_{P,mix}v_{GAS}\frac{\partial T}{\partial z}-U\frac{4}{d}\left(T-T_{e}\right)-\rho_{catalyst}\left(1-\varepsilon_{pb}\right)\sum_{j}{\Delta H}_{j}\;f\;{\eta \;r}_{j}$$

To evaluate the reaction rate, the kinetic model of Koschany et al.^35^ was considered for the methanation reaction (Meth) (eq 5). The kinetics of Xu and Froment^36^ for both the water gas shift (WGS) (eq 6) and the steam reforming (SR) (eq 7) were used in line with previous studies.^37,38^

4 $$\eta =\frac{3}{{\varphi_{s}}^{2}}\left[\varphi_{s}\coth\left(\varphi_{s}\right)-1\right]$$

$$\varphi_{s}=\frac{d_{P}}{2}\sqrt{\frac{k_{Meth}}{D_{e}}}$$

$$D_{e}=\frac{0.00143T^{1.75}{\left(\frac{1}{{M_{W}}_{{CO}_{2}}}+\frac{1}{{M_{W}}_{H_{2}}}\right)}^{0.5}}{P\sqrt{2}\left(V_{{CO}_{2}}^{1/3}+V_{H_{2}}^{1/3}\right)}$$

To further validate this hybrid approach, an additional analysis in which the WGS and SR reactions were decoupled from the methanation network was carried out. The comparison between the full kinetic scheme (Meth + WGS + SR, Figure S1a) and the simplified case that only includes methanation shows that the overall results are essentially analogous (Figure S1b). As expected, excluding the kinetics of the WGS and SR reactions results in no CO being produced along the reactor, particularly at the temperature hotspot. In contrast, in the full kinetic system, the local temperature increase near the hotspot promotes CO formation via the endothermic WGS/SR routes. The generated CO acts as a reaction intermediate and is rapidly converted into CH_4_ via the CO methanation pathway, which is more exothermic than CO_2_ methanation and speeds up the system. This is clearly demonstrated by the CH_4_ selectivity profile, which shows a distinct inflection point corresponding to the CO-mediated methanation step (*T*_MAX_ = 692 °C). This pushes the CH_4_ selectivity to 100%. Conversely, in the case of methanation kinetics alone, the CH_4_ selectivity increases more gradually to its maximum, and the temperature profile shows slightly slower hotspot dissipation (*T*_MAX_ = 696 °C). These results confirm that including the WGS and SR reactions only has a secondary effect on the global reactor behavior without compromising thermodynamic consistency.

5 $$r_{Meth}=\frac{k_{Meth}p_{H_{2}}^{0.5}p_{CO_{2}}^{0.5}\left(1-\frac{p_{CH_{4}}p_{H_{2}O}^{2}}{p_{CO_{2}}p_{H_{2}}^{4}K_{eq,Meth}}\right)}{{\left(1+K_{mix}p_{CO_{2}}^{0.5}+K_{H_{2}}^{0.5}p_{H_{2}}^{0.5}+\frac{K_{OH}p_{H_{2}O}}{p_{H_{2}}^{0.5}}\right)}^{2}}$$

$${CO}_{\left(g\right)}+H_{2}O_{\left(g\right)}⇌{CO}_{2\left(g\right)}+H_{2\left(g\right)},{\Delta H}_{WGS}^{0}=-41kJ/mol$$

6 $$r_{WGS}=\frac{\frac{k_{WGS}}{p_{H_{2}}}\left(p_{CO}p_{H_{2}O}-\frac{p_{CO_{2}}p_{H_{2}}}{K_{eq,WGS}}\right)}{{\left(1+{\;K}_{CO}p_{CO}+K_{H_{2}}p_{H_{2}\;}+K_{CH_{4}}p_{CH_{4}\;}+\frac{K_{H_{2}O}p_{H_{2}O}}{p_{H_{2}}}\right)}^{2}}$$

$${CH}_{4\left(g\right)}+H_{2}O_{\left(g\right)}⇌{CO}_{\left(g\right)}+{3H}_{2\left(g\right),}{\Delta H}_{SR}^{0}=+206kJ/mol$$

7 $$r_{SR}=\frac{\frac{k_{SR}}{p_{H2}^{2.5}}\left(p_{CH_{4}}p_{H_{2}O}-\frac{p_{CO}p_{H2}^{3}}{K_{eq,SR}}\right)}{{\left(1+K_{CO}p_{CO}+K_{H_{2}}p_{H_{2}}+K_{CH_{4}}p_{CH_{4}}+\frac{K_{H_{2}O}p_{H_{2}O}}{p_{H_{2}}}\right)}^{2}}$$

The decrease in the catalyst active phase with a material with the same thermal properties was considered by introducing the parameter *f* (eq 8), which considers the reduction in the reaction rate by the decrease in the amount of Ni in the catalyst pellet.

8 $$f=\frac{r_{j}\left(actual\;load\;of\;the\;Ni\;catalyst\right)}{r_{j}\left(reference\;load\;of\;the\;Ni\;catalyst\right)}$$

The parameter *f* decreases the reaction rate of all of the reactions considered equally, as it represents the presence of a lower amount of Ni in the catalytic pellets. The catalyst dilution with a material with different thermal properties was accounted for by introducing Φ (eq 9), which is defined as the ratio of the catalyst volume over the reactor bed volume, constituted by the catalyst and the inert.

9 $$\rho_{bed}=\rho_{inert}\left(1-\Phi \right)+\rho_{catalyst}\Phi ,where\;\Phi =\frac{catalyst\;volume}{\left(catalyst+inert\right)volume}$$

The *f* parameter will be varied only at a constant Φ parameter to gain insights into the maximum temperature profile.

The thermal conductivity of the bed (λ_bed_) varies between 1 and 250 W/(m °C) to take into account several inert materials of industrial interest, according to (eq 10).

10 $$\lambda_{bed}=\lambda_{inert}\left(1-\Phi \right)+\lambda_{catalyst}\Phi$$

According to the formulation proposed by Tsotsas,^31,39^ the effective thermal conductivities of the gas and solid phases are evaluated according to (eq 11).

Although ρ_bed_ and Φ do not explicitly appear in the mass and energy balance equations (eqs 2 and 3), both are used to estimate the heat transfer properties of the reactor. In particular, they are used to evaluate the Nusselt number (eq 12) and, consequently, the wall heat transfer coefficient (eq 13), thereby influencing the overall heat transfer of the reactor.

11 $$\frac{\lambda_{bed,eff}}{\lambda_{g}}=1-\sqrt{1-\Psi }-k_{c}\sqrt{1-\Psi }$$

$$k_{c}=\frac{2}{N}\left(\frac{B}{N^{2}}\frac{k_{p}-1}{k_{p}}\;\ln\;\frac{k_{p}}{B}-\frac{B+1}{2}-\frac{B-1}{N}\right)$$

$$N=1-\frac{B}{k_{p}},,k_{p}=\frac{\lambda_{bed}}{\lambda_{g}},,B=1.25{\left(\frac{1-\Psi }{\Psi }\right)}^{10/9}$$

The thermal conductivity of the bed was used to evaluate the wall heat transfer coefficient (α_wall_), according to the Martin and Nilles correlation for a fixed bed reactor (eqs 12 and 13).^31,40^ This is based on stagnant and dynamic contributions, considering the conductivity of the solid and gas phases.

12 Nu=(1.3+5dtubedp)λbed,effλg+0.19Re0.75Pr0.33

13 $$Nu=\frac{\alpha_{wall\;}d_{tube}}{\lambda_{g}}$$

Finally, to calculate the overall heat transfer coefficient, the boiling water heat transfer coefficient was evaluated as a function of wall thickness, according to the equation (eq 14), where *k*_water_ is set equal to 3000 W/(m^2^ K).

14 $$U=\frac{1}{\left(\frac{1}{\alpha_{wall}}+\frac{1}{\alpha_{water}}\right)},where\;\frac{1}{\alpha_{water}}=\frac{s}{100}+\frac{1}{k_{water}}$$

The list of possible inert materials is reported in Table 2, along with the most relevant parameters.

**Table 2:** List of the Considered Inert Materials with Their Respective Densities and Conductivities (λ_inert_) and the Thermal Conductivity of the Catalytic Bed at 25 °C for Φ = 0.3 (λ_bed_)^41–43^

inert material	solid density [kg/m^3^]	thermal conductivity of inert material [W/(m °C)]	thermal conductivity of catalytic bed for Φ = 0.3 [W/(m °C)]
SiO_2_	2300	1	1.0
CeO_2_	7650	2	1.7
ZrO_2_	5850	5	3.8
TiO_2_	4000	7	5.2
SrTiO_3_	5100	12	8.7
Y_2_O_3_	5000	25	17.8
Al_2_O_3_	4000	30	21.3
ZnO	5600	60	42.3
SiC	3200	150	105.3
BN	2200	250	175.3

## Results and Discussion

### Model Validation

As a first step, the developed model was validated against experimental data. To this end, the results by Dannesboe et al.^30^ were considered, and the model was applied according to the dimensions provided in the reference and cited above. The processed biogas had a composition of 40% CO_2_ and 60% CH_4_, with a total flow rate of 10 N m^3^/h. The reactor was operated with cooling water circulated at 280 °C and a biogas pressure of 21 bar. Figure 1 illustrates the simulation results and their comparison to the experimental data points. A quantitative comparison between the model and experimental temperature profiles^30^ was performed at 21 bar. The root-mean-square error (RMSE) was 25.8 °C, and the maximum deviation was less than 48 °C. The fit of the model to the experimental data points is good, and the model describes well the cooling phase of the reactor after the reaction hotspot. It is hence highly likely that the model developed can perform a good prediction of the cooling properties of the reactor in an identical geometrical configuration. As at 21 bar, the maximum hotspot is above 650 °C; it was decided to analyze the reaction at lower pressure so that the maximum hotspot temperature is lower. The selected pressure for the rest of the study is 8 bar, which was found as a feasible pressure level to produce an SNG with sufficient quality for grid injection.^8^ The choice of a lower pressure should not significantly change the prediction power of the model, as the main effects of pressure are on the thermodynamic equilibrium conversion (CO_2_ adiabatic conversion decreases from 77% at 21 bar to 73% at 8 bar, with a decrease in the adiabatic temperature from 670 to 580 °C). In any case, the effect of the pressure on the plant was investigated and will be shown later. One can observe that the temperature of the hotspot in the pilot reactor reaches a significant peak near the reactor inlet. This is due to the large amount of heat produced by the rapid reaction of H_2_ and CO_2_. This phenomenon should be limited to preventing damage to the catalyst, as highlighted above. Therefore, the operating conditions must be set appropriately to avoid the instauration of uncontrolled temperature hotspot conditions.^31,44,45^ The first calculations showed that the reaction rate at 21 bar is too high to allow the control of the reaction in a fixed-bed reactor. For this reason, the operating pressure was reduced from 21 bar (validation data) to 8 bar to limit both the reaction rate and the hotspot temperature, thus facilitating the management of the process parameters. The predicted decrease in the hotspot temperature when the pressure was reduced from 21 to 8 bar has been evaluated to be close to 70 °C, as shown in Figure 1, which is in line with the predicted decrease of the adiabatic temperature. This decrease in hotspot temperature with pressure suggests that the system can be better controlled by selecting the appropriate control parameters. The next option to further reduce the hotspot temperature is to dilute the catalyst, which will be described in the next section by exploring the effect of the Φ parameter first and then evaluating the effect of *f* at a constant Φ.

**Figure 1:**
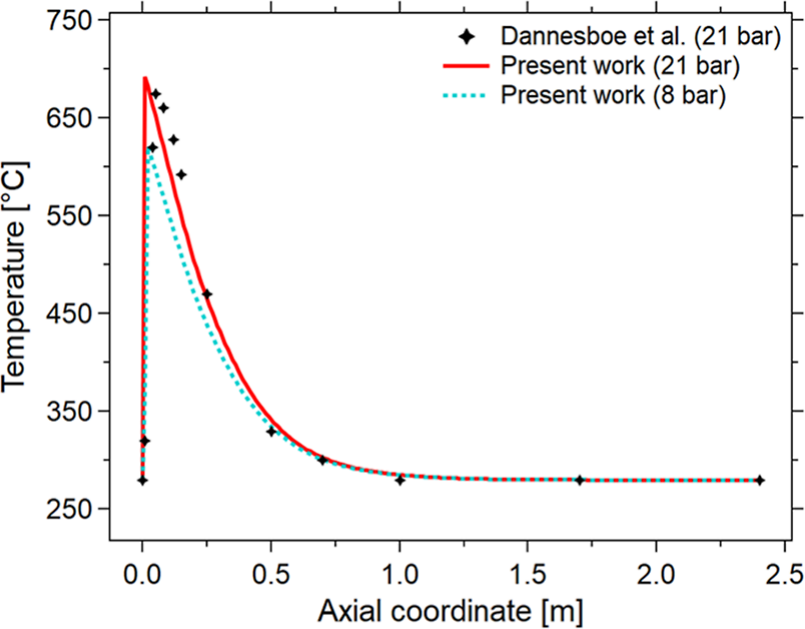
Validation of the data obtained in the present work against experimental data. Temperature [°C] vs axial coordinate [*m*] for different pressures. Experimental conditions: CO_2_/CH_4_/H_2_ = 1/1.5/4, total flow rate equal to 10 Nm^3^/h, *T* = *T*_e_ = 280 °C (the experimental data are reported by Dannesboe et al.^30^).

Additionally, the validation of the model has been carried out also on experimental data obtained with a different reactor geometry^8^ operating at 8 bar in a large-scale direct biogas methanation system. The results are reported for the sake of comparison in the Supporting Information (Figure S2).

### Effect of Catalyst Dilution with Selected Inert Materials

To enlarge the understanding of the effect of dilution further, the variation of the maximum temperature and CO_2_ conversion as a function of the dilution of the catalyst (Φ) was evaluated in Figure 2. The system’s performance is significantly affected by the conductivity of the inert material, underscoring the importance of selecting it carefully. When using SiC or BN, characterized by a very high λ_inert_, the reactor operates in a low-temperature regime, while for SiO_2_, CeO_2_, and ZrO_2_, a remarkable temperature increase is observed. As the thermal conductivity of the bed is too high, there is a shift toward higher Φ in the CO_2_ conversion profiles, and the simulated hotspot temperature is much lower than in other cases. This means that the reactor operates in a low-temperature regime, and therefore, there is a remarkable kinetic limitation. Thus, controlling the hotspot temperature and the amount of heat generated is crucial to prevent excessive temperatures, which can reduce the lifespan of the catalyst. The choice of an inert material with too-low thermal conductivity leads to a remarkable increase in the hotspot temperature, causing the production of CO, which is a poison for many catalysts.^46^ This is in line with what has already been observed in the literature.^47^ It is therefore necessary to choose an inert material with proper thermal conductivity to better control the temperature in the reactor without causing a rapid increase in the maximum temperature profile. In the present work, 550 °C was set as the maximum acceptable hotspot temperature (to avoid compromising the Ni-based catalyst) with a minimum acceptable CO_2_ conversion of 97% (minimum conversion required for grid injection in many European countries).^48^ Consequently, in the present case, only the use of Y_2_O_3_, Al_2_O_3,_ ZnO, SiC, and BN (each in the proper Φ range between 0.2 and 0.9) apparently allows the design of a highly efficient reactor. Indeed, despite the set CO_2_ conversion is easily achieved by all the materials for Φ ≥ 0.2, the maximum temperature of 550 °C is overcome by SiO_2_, CeO_2_, ZrO_2_, TiO_2_, and SrTiO_2_ already for Φ ≥ 0.1. Therefore, the hotspot temperature can be effectively considered a critical parameter.

**Figure 2:**
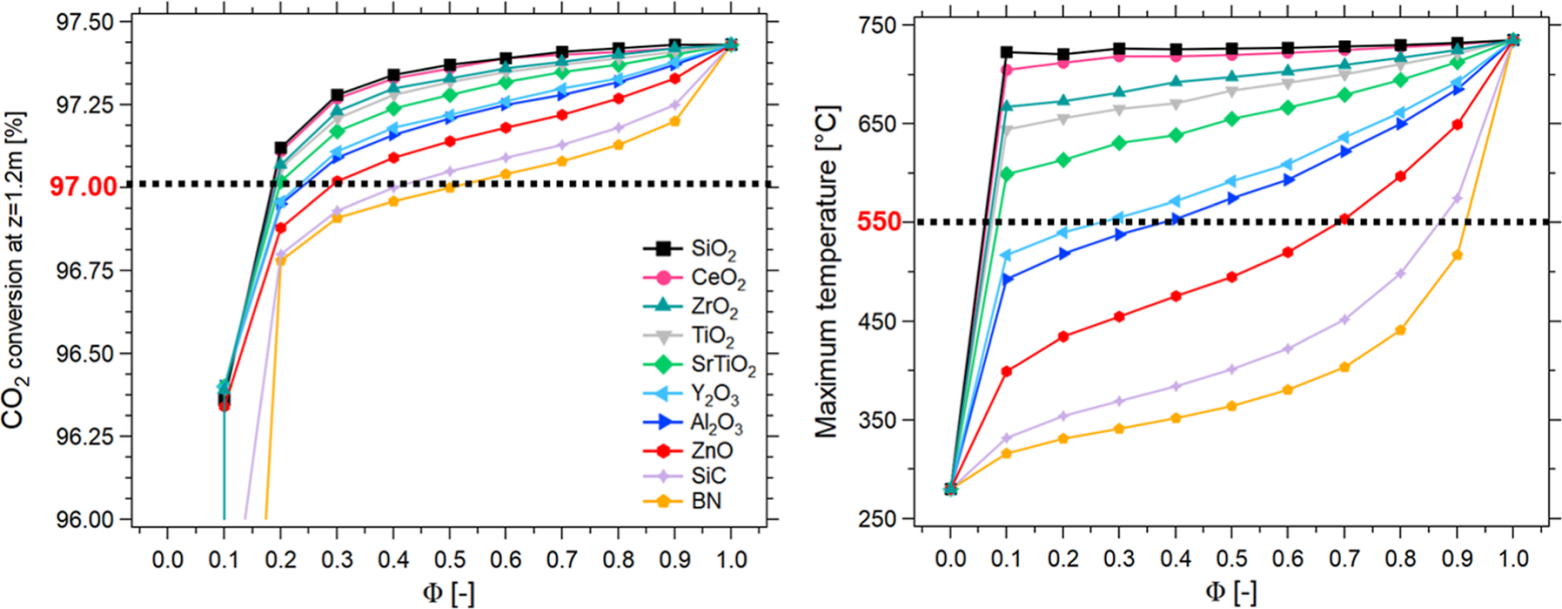
Simulation of biogas methanation reactor—CO_2_ conversion at *z* = 1.2 m [%] (left) and maximum temperature [°C] (right) vs catalytic dilution coefficient Φ [-] for different inert materials (symbols). Experimental conditions: CO_2_/CH_4_/H_2_ = 1/1.5/4, total flow rate equal to 5 Nm^3^/h, *p* = 8 bar, *T* = *T*_e_ = 280 °C.

However, the use of highly conductive dilutants forces the system to operate in a low-temperature regime, as shown in Figure 3. This results in the requirement of utilizing low GHSV (large reactor volume) to reach the target conversion. Indeed, the SiC profile shows a constant low-temperature regime at almost all dilution factors. This allows for achieving CO_2_ conversion above 97% only when Φ > 0.4. Therefore, it is difficult to reach the required conversion with a reasonable reactor size. On the other hand, the use of yttria, alumina, or zinc oxide allows controlling the temperature increase around the desired CO_2_ conversion, as the maximum temperature rises gradually and mildly, up to a sudden increase to high values for Φ approaching unity. Hence, it is possible to find a range of Φ values sufficiently large for practical application. For these materials, a catalytic formulation with a dilution factor between 0.3 and 0.6 can results in a significant improvement in the CO_2_ methanation performance, meeting the hotspot temperature and CO_2_ conversion requirements.

**Figure 3:**
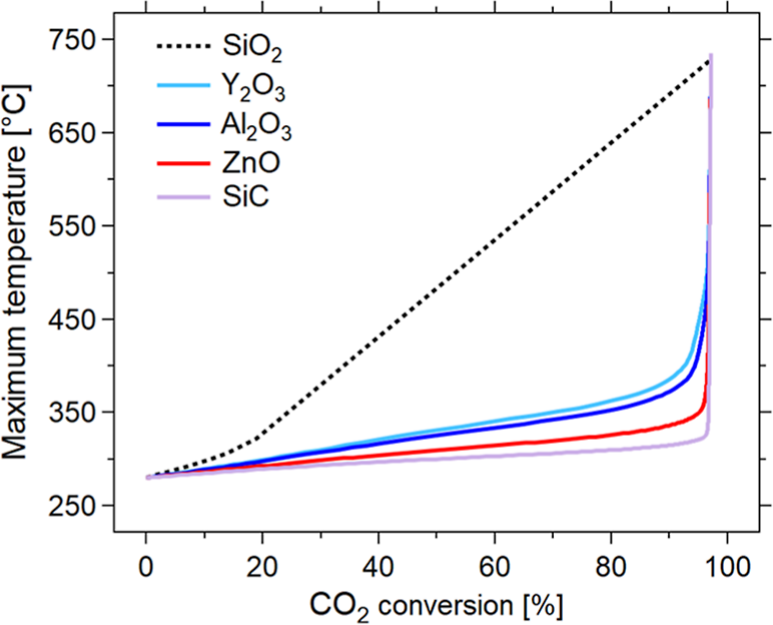
Simulation of biogas methanation reactor—maximum temperature [°C] vs CO_2_ conversion at *z* = 1.2 m [%] for different inert materials (colors), varying catalytic dilution coefficient Φ between 0.1 and 1­[-]. Experimental conditions: CO_2_/CH_4_/H_2_ = 1:1.5:4, total flow rate equal to 5 Nm^3^/h, *p* = 8 bar, *T* = *T*_e_ = 280 °C.

Figure 4 shows the variation of the maximum temperature and CO_2_ conversion as a function of the chosen inert material, according to the λ_bed_ formulation (eq 10). In this case, a catalytic dilution factor (Φ) of 0.3 was preliminarily set based on the above-discussed results. These plots confirm that the choice of yttria, alumina, and zinc oxide ensures that the reactor is kept under favorable operating conditions. Indeed, although slightly higher CO_2_ conversions are achieved with the low thermal conductivity materials, the hotspot temperatures are significantly higher, with temperatures up to ∼750 °C. The achievement of higher hotspot temperatures, always below the desired threshold, allows better exploiting the kinetics of the reaction, allowing to reach the target conversion with significantly higher GHSV. This confirms what has already been observed in previous studies for the Ru-based catalyst.^49^ In this sense, SiC and BN would require too low of a GHSV to be used in practical applications.

**Figure 4:**
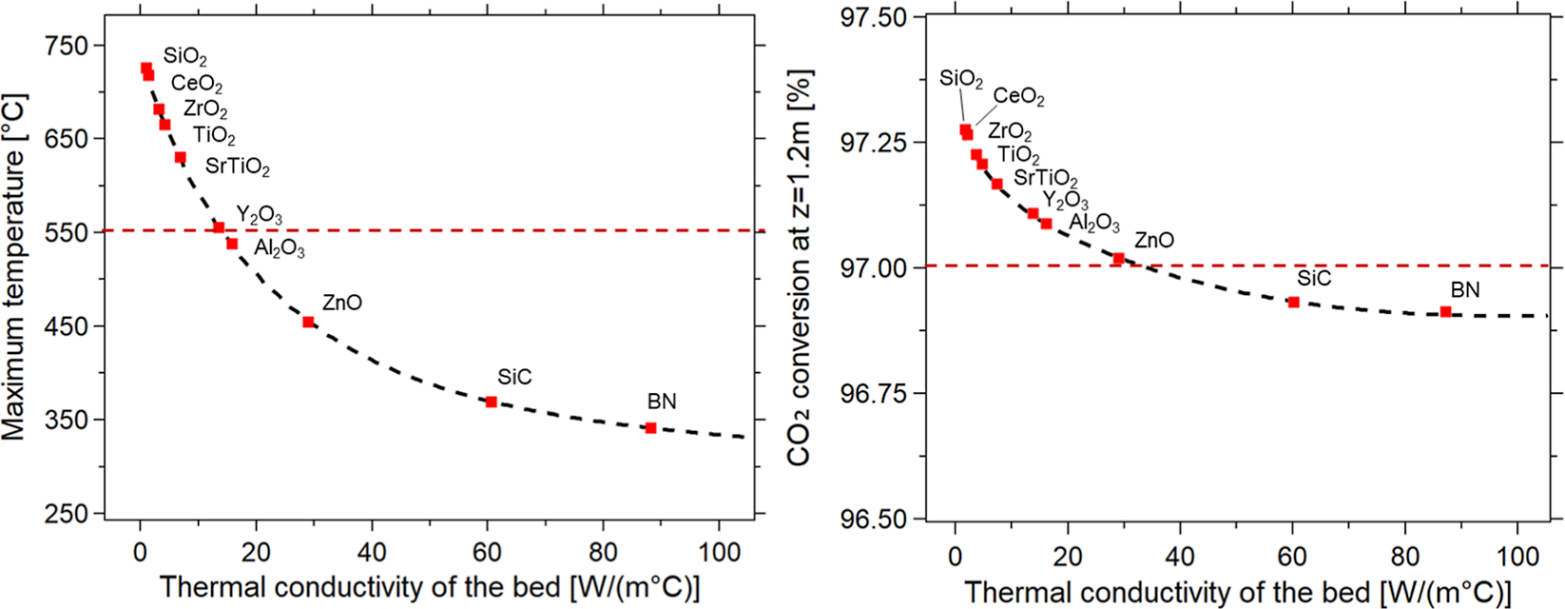
Simulation of biogas methanation reactor—maximum temperature [°C] (left) and CO_2_ conversion at *z* = 1.2 m [%] (right) vs thermal conductivity of the bed λ_bed_ [W/(m °C)] for different inert materials. Experimental conditions: CO_2_/CH_4_/H_2_ = 1:1.5:4, total flow rate equal to 5 Nm^3^/h, *p* = 8 bar, *T* = *T*_e_ = 280 °C, Φ = 0.3 (thermal conductivity calculated according to (eq 9)).

### Effect of Pressure

The effect of pressure on the reaction system is investigated in Figure 5; the variations in CO_2_ conversion and maximum temperature have been analyzed as a function of catalyst dilution (Φ). The effect of heat release and reactor cooling was evaluated for three different scenarios based on the thermal conductivity of the inert materials, namely, SiO_2_, Al_2_O_3_, and SiC. Points above the dilution factor that causes the reach of too high temperature are omitted from the graph to favor the readability. In all cases, operating at atmospheric pressure enables the temperature profile to be easily controlled. However, significant limitations in the CO_2_ conversion were observed, with values 5–6% lower than at higher pressures. Moreover, operating at high pressure (20 bar) enabled achieving high CO_2_ conversion (>97%), but temperature control was challenging, particularly at high thermal conductivity of the packed bed. Furthermore, the lower the thermal conductivity of the inert material, the higher the maximum temperature that can be achieved. This results in a difficult control of the temperature, that, for example, can reach critically high values on SiO_2_, even concentrating the heat production over a small fraction of the reactor, hence reaching higher values than those observed by Dannesboe et al.^30^ Hence, at high pressure, the use of an inert material with high heat conductivity is more appropriate than in the low-pressure case. At 20 bar, dilution with SiC could be an affordable solution to control the temperature while achieving high conversion with a practically acceptable GHSV. Looking at an inert material with intermediate thermal conductivity, such as Al_2_O_3_, leads to a proper balance of heat release and cooling of the reaction system at up to 10 bar. However, operating at high pressures (>10 bar) and with low catalyst dilution (Φ > 0.8) can lead to an uncontrolled temperature hotspot. Additionally, controlling the catalyst dilution values is necessary to maintain an operating temperature below 550 °C and preserve the catalyst activity. Therefore, it is desirable to limit the reaction pressure to 10 bar to ensure that the temperature hotspot can be controlled, unless the catalyst is diluted with highly conductive materials like SiC. This confirms what is qualitatively underlined in the previous sections. However, the operation at higher pressure and hence at higher reaction rate favors the use of dilutant with higher conductivity to reduce the overall reaction rate and better dissipate the heat of reaction.

**Figure 5:**
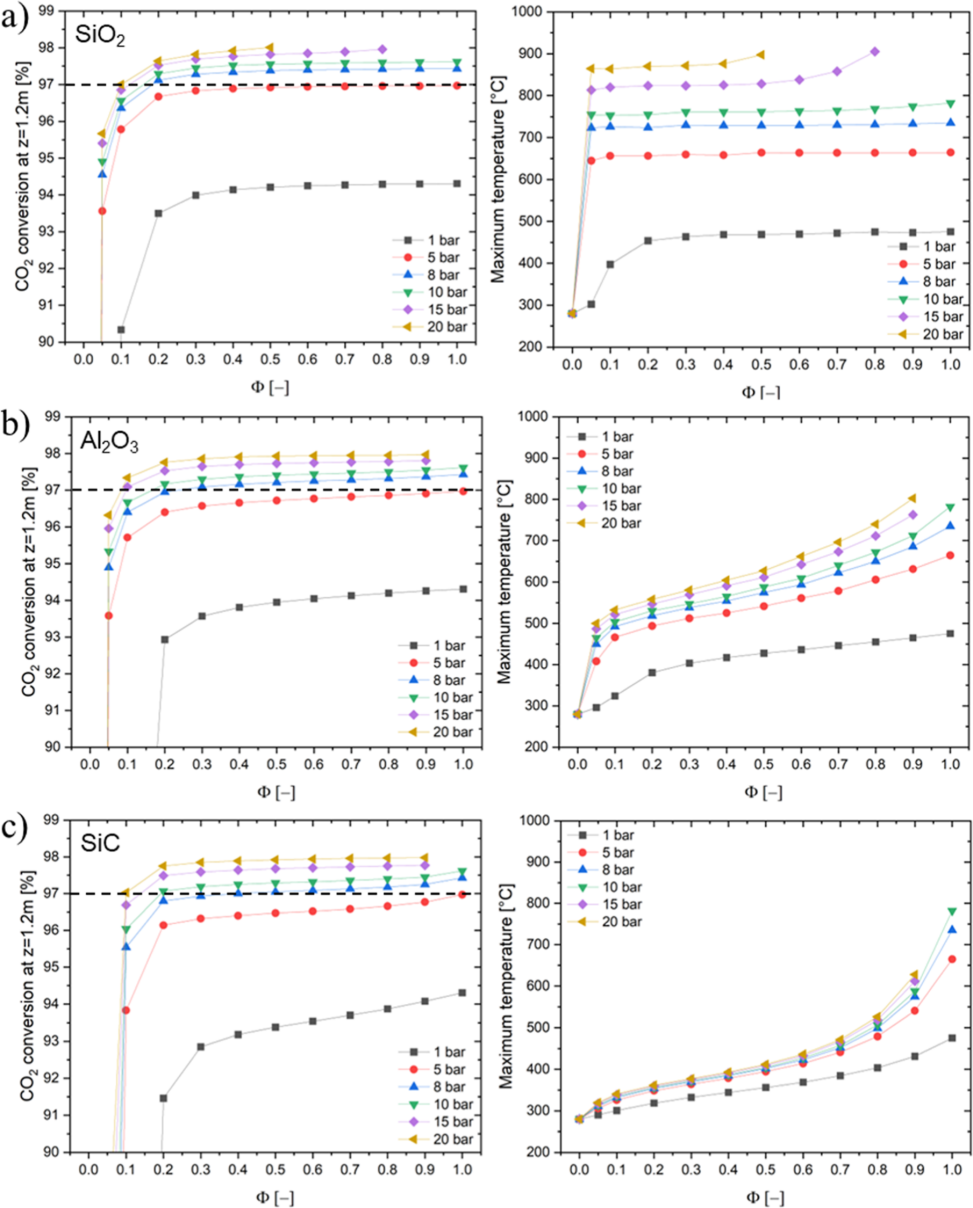
Simulation of biogas methanation reactor—CO_2_ conversion at *z* = 1.2 m [%] (left) and maximum temperature [°C] (right) vs catalytic dilution coefficient Φ [-] for Ni/Al_2_O_3_ diluted with SiO_2_ (a), Al_2_O_3_ (b), and SiC (c) catalyst varying the operating pressure (symbols, 1, 5, 8, 10, 15, and 20 bar). Experimental conditions: CO_2_/CH_4_/H_2_ = 1:1.5:4, total flow rate equal to 5 Nm^3^/h, *T* = *T*_e_ = 280 °C.

### Effect of the Load of the Active Phase

The reduction of the active catalytic material amount allows a decrease in the reaction rate; however, it does not reduce the tendency of the system to be subject to parametric sensitivity. This is highlighted by the results of the simulations displayed in Figure 6, where the temperature profile and the maximum temperature were controlled by varying the parameter *f* (eq 8), which introduces a dilution effect by considering Al_2_O_3_ as an inert material, based on the previously obtained results.

**Figure 6:**
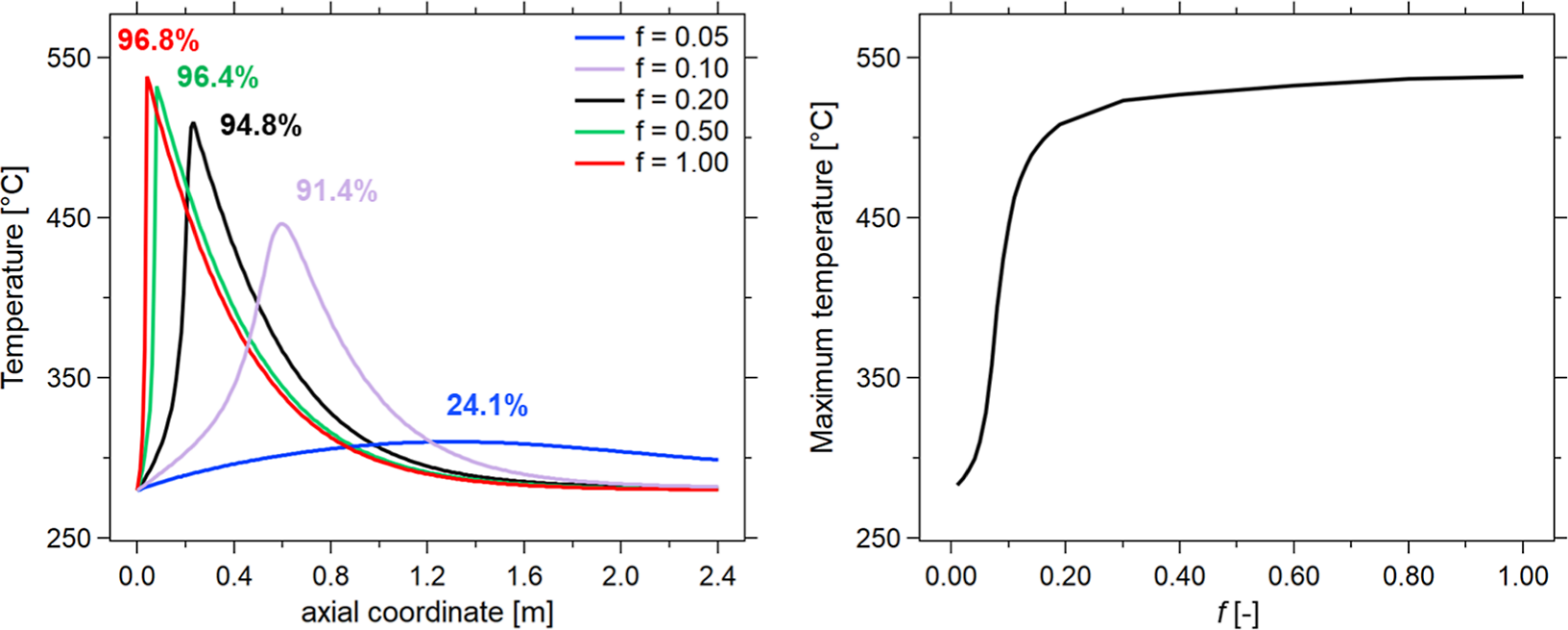
Effect of the reduction of catalytic material (*f* = reaction rate of diluted catalyst/reaction rate of the reference catalyst)—temperature [°C] vs axial coordinate [m] (left) for different reaction rate values and corresponding CO_2_ conversions (colors) —maximum temperature [°C] vs reaction rate values [-] (right). Experimental conditions: CO_2_/CH_4_/H_2_ = 1/1.5/4, total flow rate equal to 5 Nm^3^/h, *p* = 8 bar, *T* = *T*_e_ = 280 °C, Φ = 0.3.

A reduction of the reaction rate ratio up to 50% (*f* = 0.5) does not have a significant effect on the CO_2_ conversion and temperature hotspot, in terms of both position and values achieved. This is due to the considerable reaction rate in the high-temperature region, which causes a large amount of heat production even when halving the concentration of the active material. An effective reduction of the CO_2_ conversion and hotspot temperature is possible only below a *f* factor of 0.10, where the modeled CO_2_ conversion and temperature reach a maximum of 91.4% and 450 °C, respectively, which then decrease and broaden upon further decrease of *f*. However, under these conditions, the reaction rate is low, and the conversion at the reactor outlet is significantly reduced compared to previous cases, resulting in a stream that is not suitable for direct grid injection. As evident in Figure 6, a discontinuity exists between configurations that operate with a reaction hotspot and those with low-temperature operation. This is a clear example of the parametric sensitivity to which CO_2_ methanation is subject. The two different regimes are distinguished into two opposite cases: one case where the reaction rate and the heat production trigger each other, causing the formation of the temperature hotspot, and one case where the reaction rate is never sufficiently high to cause the increase of temperature and the consequent further increase of the reaction rate itself.

### Effect of Different Inert Materials on a Φ = 0.3 Packed Bed Reactor

To mitigate the effect of parametric sensitivity, it is necessary to modify the reactor properties either to distribute the heat production over a larger space or to reduce the reaction rate independently of the heat production. A value of UA_sez_ of 600 W/°C was assumed from the estimated overall heat transfer coefficient under the operating conditions of the reactor studied. Sensitivity tests (Figure S3) confirmed that variations in this parameter did not significantly affect the temperature profiles or conversion, thereby supporting the robustness of the chosen value. A wide range of thermal conductivities of the packed bed (λ_bed_) was investigated. The effect of selected inert materials, with a constant Φ of 0.3, has been examined, and the results are shown in Figure 7. It can be observed that the nature of the inert state has a more relevant effect on the temperature than the dilution itself (compared with Figure 6). The use of an inert material with higher conductivity results in a more uniform temperature profile along the reactor axis, while decreasing the maximum temperature reached. This reduces thermal gradients and the risk of localized hotspots, leading to more controllable and thermally stable operations. As reported in Figure 7, the high thermal conductivity will change the heat dissipation in the hotspot region, allowing it to reach it in the first part of the reactor. Using materials with lower thermal conductivity, such as SiO_2_ and Al_2_O_3_, enables the heat to be removed within the first 0.5 min of the reactor. Inert materials characterized by higher conductivity can eventually cause the reactor to operate in a low-temperature regime, as observed for SiC. For this reason, materials like SiC are commonly used as dilutants for lab-scale kinetic experiments to ensure isothermal conditions.^50^

**Figure 7:**
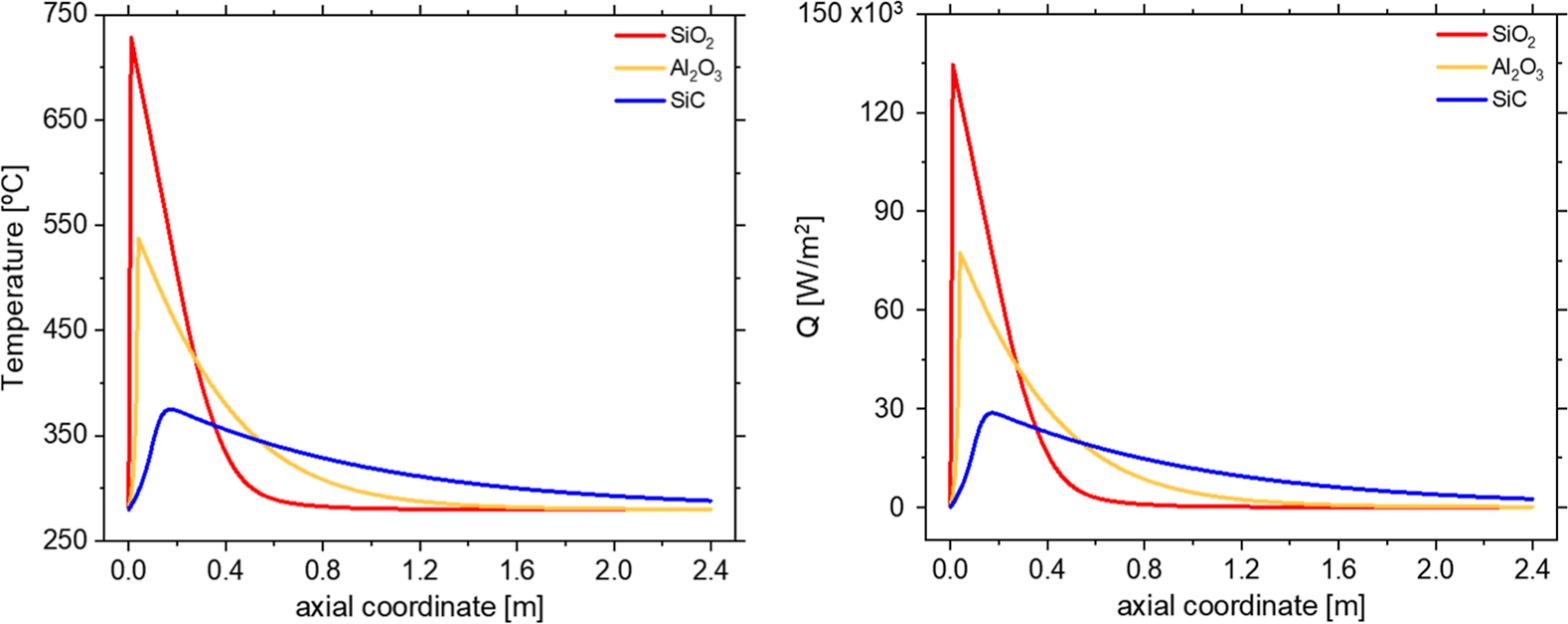
Simulation of biogas methanation reactor—temperature [°C] (left) and heat transfer rate [W/m^2^] (right) vs axial coordinate [*m*] for different inert materials (colors) at a constant UA_sez_ = 600 W/°C. Experimental conditions: CO_2_/CH_4_/H_2_ = 1/1.5/4, total flow rate equal to 5 Nm^3^/h, *p* = 8 bar, *T* = *T*_e_ = 280 °C, Φ = 0.3.

### Multilayer Reactor

It was observed so far that one single catalyst layer with a specific dilution is not sufficient to achieve optimal results, as the trade-off between temperature hotspot and achievable conversion is difficult to break. Therefore, it is worth discussing the option of utilizing a multilayer reactor to control the heat release and the reactor cooling. This reactor configuration consists of two sequential steps with different catalytic inert materials.^51^ The switch between the two sequential steps is based on temperature values and is set in the Matlab R2023a code (Figure 8). The first section of the reactor is crucial because it includes hotspot formation. Therefore, based on previous results, an alumina-supported catalyst with a dilution factor of 0.3 (Φ) was assumed to keep the maximum temperature below 550 °C. The switch from the alumina-supported to the silica-supported step of the reactor bed then allows the temperature profile to be optimized. Consequently, the multilayer reactor returned to the coolant temperature more rapidly than with an alumina-only catalyst. Furthermore, the coolant temperature is reached in a smaller portion of the catalytic bed than in the single layer reactor. This concept can significantly reduce the volume required to reach steady-state conditions at a stable working point. This can give significantly larger advantages than a multilayered methanation reactor with different catalysts,^52^ as we demonstrated that the most critical parameter is heat conductivity rather than the catalyst activity. Hence, this is a promising option to break the trade-off between achievable conversion and temperature hotspot. However, it should be underlined that this advantage is obtained at the cost of lower flexibility of the reactor, as a change in the axial profiles due to load changes may cause the instauration of less favorable conditions (with the catalyst layer change resulting in a non-ideal position).

**Figure 8:**
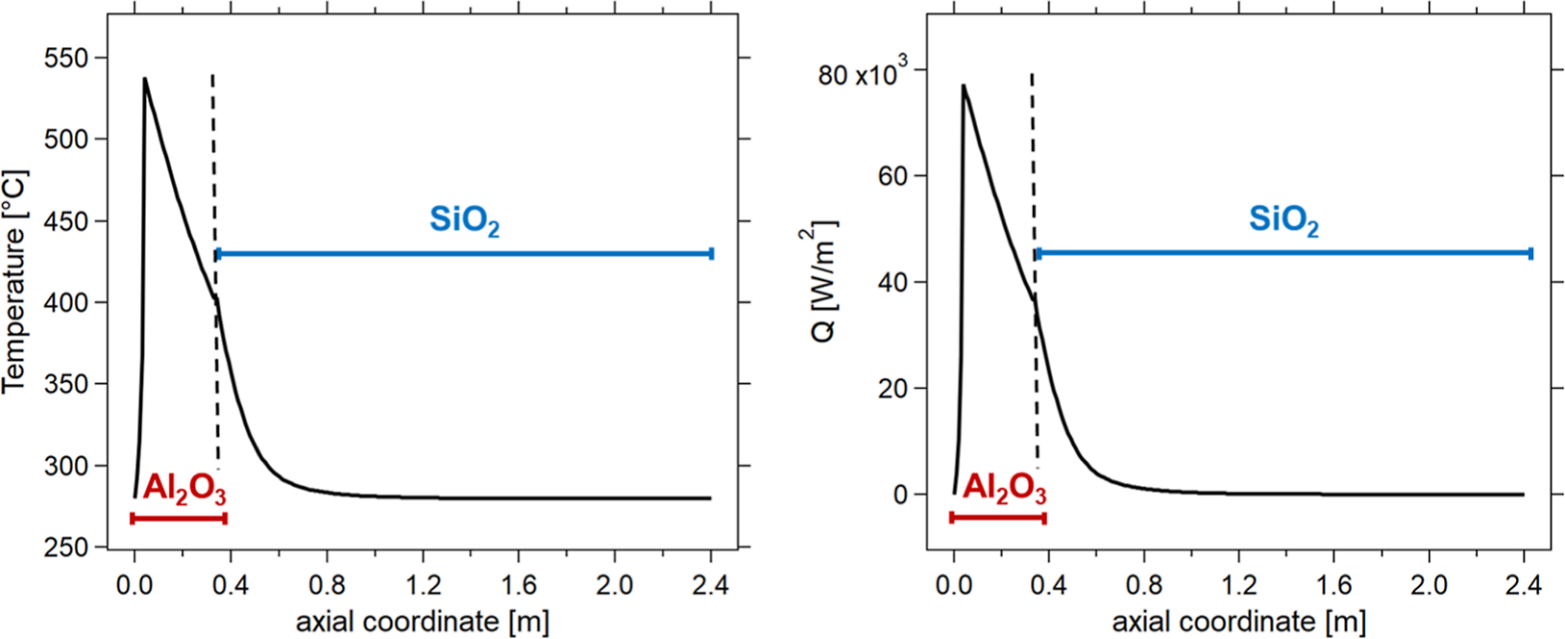
Flexible reactor—temperature [°C] (left) and heat transfer rate [W/m^2^] (right) vs axial coordinate [*m*] for multilayer reactor (colors). Experimental conditions: CO_2_/CH_4_/H_2_ = 1/1.5/4, total flow rate equal to 5 Nm^3^/h, *p* = 8 bar, *T* = *T*_e_ = 280 °C, Φ = 0.3.

## Conclusions

This work demonstrated how an improved design of the coupling catalyst/inert material can enhance the performance of biogas methanation reactors, ensuring safe and economically viable operation in terms of hotspot temperature, reaction yield, and reactor dimensions. The methodology developed is based on the use of catalyst inert agents tailored to specific properties and amounts to achieve the desired output (reduction of the hotspot) while maintaining the required productivity of the reactor. The main aim of the study was the rupture of the trade-off between reaction hotspot and CO_2_ conversion.

In the context of biogas methanation, many diluting materials were examined, leading to the development of a significant data set reporting under which conditions the temperature hotspot is expected to surpass the critical threshold of 550 °C. It was observed that materials with a conductivity below 10 W/(m °C) are not effective in spreading the heat production over the axial coordinate of the reactor; hence, they do not serve to improve the operability of the reactor. On the other extreme, materials with a conductivity above 100 W/(m °C) are too effective in heat dissipation, so diluting the catalyst with these materials causes the operation to occur in a low-temperature regime, requiring a low GHSV to achieve high CO_2_ conversion. Y_2_O_3_, Al_2_O_3_, and ZnO appeared as ideal candidates for designing a reactor that can achieve high CO_2_ conversion with high GHSV and without exceeding the critical temperature of 550 °C. It was observed that the most suitable reactor configuration can be designed by using one of the latter materials as a dilutant for the Ni-based catalyst with an appropriate total thermal conductivity.

This work highlighted that the rational design of the catalyst formulation has high potential in breaking the trade-off between reactor performance and hotspot temperature in biogas methanation reactors. However, the methodology defined here can, in principle, be utilized for any exothermic reaction, hence showing the potential to improve the reactor design for several CO_2_ utilization reactions. Additionally, the case presented here did not show the effect of the temperature profile adjustment on selectivity as the selectivity to methane over the considered reactor is high. Nevertheless, the application of the method to reactions affected by selectivity problems caused by imprecise control of the temperature can lead to significant advancements in terms of selectivity improvement, with potentially evident economic advantages.

## Supplementary Material



## Notes

CO_2_ conversion; validation of the model; and simulation of biogas methanation reactor (PDF)

## Nomenclature

*A*_sez_: area of the reactor section [m^2^]
*C*_P,mix_: heat capacity of the gas mixture [J/(kg °C)]
*d*: internal reactor diameter [m]
*d*_P_: particle diameter [m]
*d*_tube_: tube diameter [m]
*D*_e_: binary diffusivity [cm^2^/s]
*f*: ratio of the reaction rate at the actual Ni loading to that at the reference Ni loading [-]
*F*_*i*_: molar flow rate of component *i* [mol/s]
GHSV: gas hourly space velocity [h^–1^]
HHV: higher heating value [kWh/Nm^3^]
i: component (CO_2_, H_2_, CH_4_, H_2_O, CO)
*j*: reaction (Meth, WGS, SR)
*K*_eq,*j*_: equilibrium constant of *j* reaction [-]
*K*_H_2_O_: dissociative adsorption constant for H_2_O
*K*_CH4_, *K*_CO_, *K*_H2_: adsorption constant for CH_4_, CO, and H_2_ (bar^–1^)
*K*_mix_: adsorption constant for CO_2_ (bar^–0.5^)
*K*_OH_: adsorption constant for hydroxyl species (bar^–0.5^)
*k*_Meth_: rate coefficient of methanation reaction [kmol/(kg_cat_ h bar)]
*k*_WGS_: rate coefficient of water gas shift reaction [kmol/(kg_cat_ h bar)]
*k*_SR_: rate coefficient of steam reforming reaction [(kmol bar^0.5^)/(kg_cat_ h)]
*k*_water_: boiling water convective heat transfer coefficient [W/(m^2^ °C)]
*L*: reactor length [m]
MW_*i*_: Molecular weight of component *i* [g/mol]
Nu: Nusselt number [-]
*P*: pressure [bar]
*p*_*i*_: partial pressure of component *i* [bar]
Pr: Prandtl number [-]
*Q*_tot_: total flow rate [Nm^3^/h]
Re: Reynolds number [-]
*r*_*j*_: reaction rate of component *i* in reaction *j* [mol/(kg_cat_ s)]
*s*: wall thickness [m]
*T*: temperature [°C]
*T*_e_: coolant temperature [°C]
*U*: overall heat transfer coefficient [W/(m^2^ °C)]
*V*_*i*_: molar volume of component *i* [cm^3^/mol]
*v*_GAS_: gas velocity [m/s]
*X*_*i*_: conversion of component *i*, i.e. CO_2_ or H_2_ [-]
*Y*_*i*_: yield to product *i*, i.e. CO or CH_4_ [-]
*z*: axial coordinate [m]
α_wall_: wall heat transfer coefficient [W/(m^2^ °C)]
α_water_: boiling water heat transfer coefficient [W/(m^2^ °C)]
Δ*H* ^0^_R_: reaction enthalpy at 25 °C [kJ/mol]
Δ*H*_*j*_: adsorption enthalpy [kJ/mol]
ε_pb_: void fraction of the catalytic packed bed [-]
η: effectiveness factor [-]
λ_bed_: thermal conductivity of the bed [W/(m °C)]
λ_bed,eff_: combined effective thermal conductivities of the gas and solid phases [W/(m °C)]
λ_g_: thermal conductivity of the gas [W/(m °C)]
λ_inert_: thermal conductivity of the inert material [W/(m °C)]
λ_P_: heat axial dispersion coefficient [W/(m °C)]
ν_*i,j*_: stoichiometric coefficient of component *i* in reaction *j* [-]
ρ_bed_: density of the catalytic bed [kg/m^3^]
ρ_catalyst_: bulk catalyst density [kg/m^3^]
ρ_gas_: density of the reactive gas [kg/m^3^]
ρ_inert_: inert material density [kg/m^3^]
φ_s_: Thiele modulus [-]
Φ: catalytic dilution [-]
ψ: bed porosity [-]
