# Supplementary material for: Adaptation of Temperature Profiles in CO2 Methanation Reactors by an Appropriate Selection of Catalyst and Dilution Agent
Source: Ind Eng Chem Res. 2026 Jan 21;65(4):1994–2005. doi: 10.1021/acs.iecr.5c03331 (PMC12919378; doi:10.1021/acs.iecr.5c03331)
Supplement: Supplementary file 1 [file ie5c03331_si_001.pdf]

Supplementary

Adaptation of temperature profiles in CO_2 methanation reactors by an appropriate selection of catalyst and dilution agent

Matteo Percivale [a,b], Mauro Andrea Pappagallo [b], Emanuele Moioli [b,c*], Gabriella Garbarino [a,d*]

[a] Dipartimento di Ingegneria Civile, Chimica e Ambientale, Università degli Studi di Genova, Via all'Opera Pia 15, 16145 Genova, Italy

[b] Center for Energy and Environmental Science, Paul Scherrer Institute, Forschungsstrasse 111, 5232 Villigen, Switzerland

[c] Dipartimento di Chimica, Materiali e Ingegneria Chimica 'Giulio Natta', Politecnico di Milano, Piazza Leonardo da Vinci 32, 20133 Milano, Italy

[d] INSTM, UdR Genova, Via Dodecaneso 31, 16146, Genoa, Italy

*corresponding authors: emanuele.moioli@polimi.it; gabriella.garbarino@unige.it

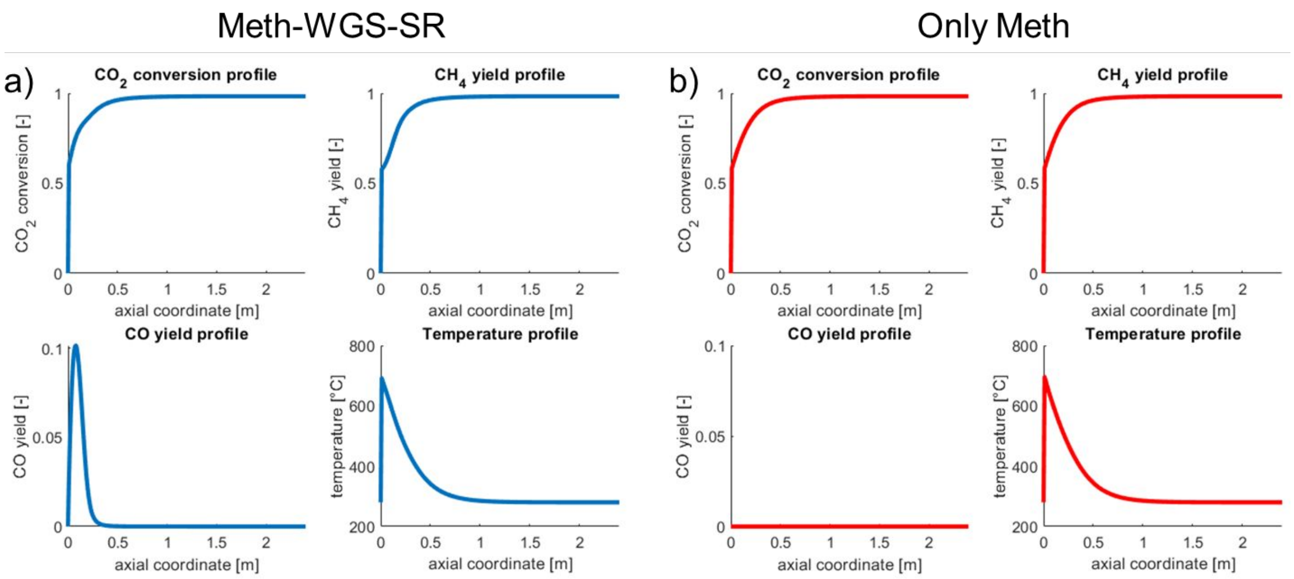

Figure S1. CO_2 conversion [-], CH_4 yield [-], CO yield [-], and temperature [°C] vs axial coordinate [m] for Meth-WGS-SR (a) and for only Meth kinetic scheme (b).

CO_2 conversion (Eq.1, X_{CO2}), and CH_4 and CO yields (Eq.2, Y_i where i = CH_4, CO) were evaluated in accordance with the following equations.

$$X_{CO2} = \frac{F_{CO2}^{in} - F_{CO2}^{out}}{F_{CO2}^{in}} \tag{1}$$

$$Y_i = \frac{F_i^{out} - F_i^{in}}{F_{CO2}^{in}} \tag{2}$$

where Fi = molar flow rate of component i, i.e., CH_4 or CO [kmol/h]

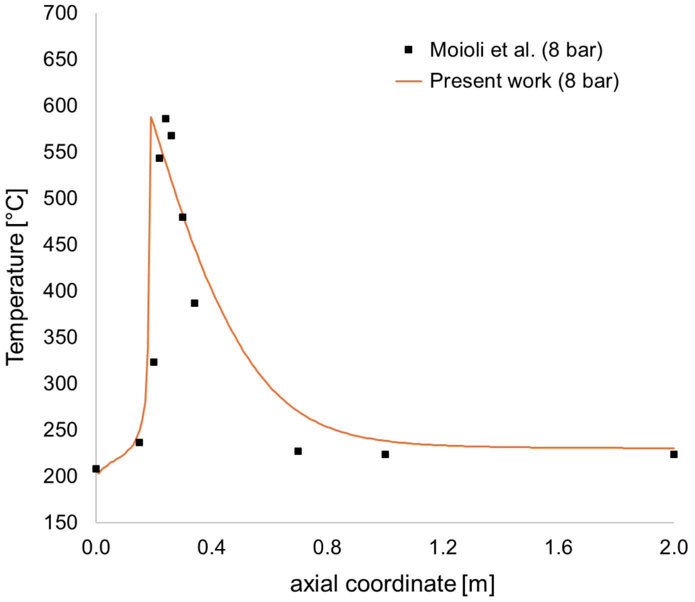

Figure S2. Validation of the model obtained in the present work against pilot plant data reported by Moioli et al. [1] - Temperature [°C] vs axial coordinate [m] at 8 bar. Experiment conditions: $CO_2/CH_4/H_2$=1/1/4.15, T=200°C, T_e=230°C.

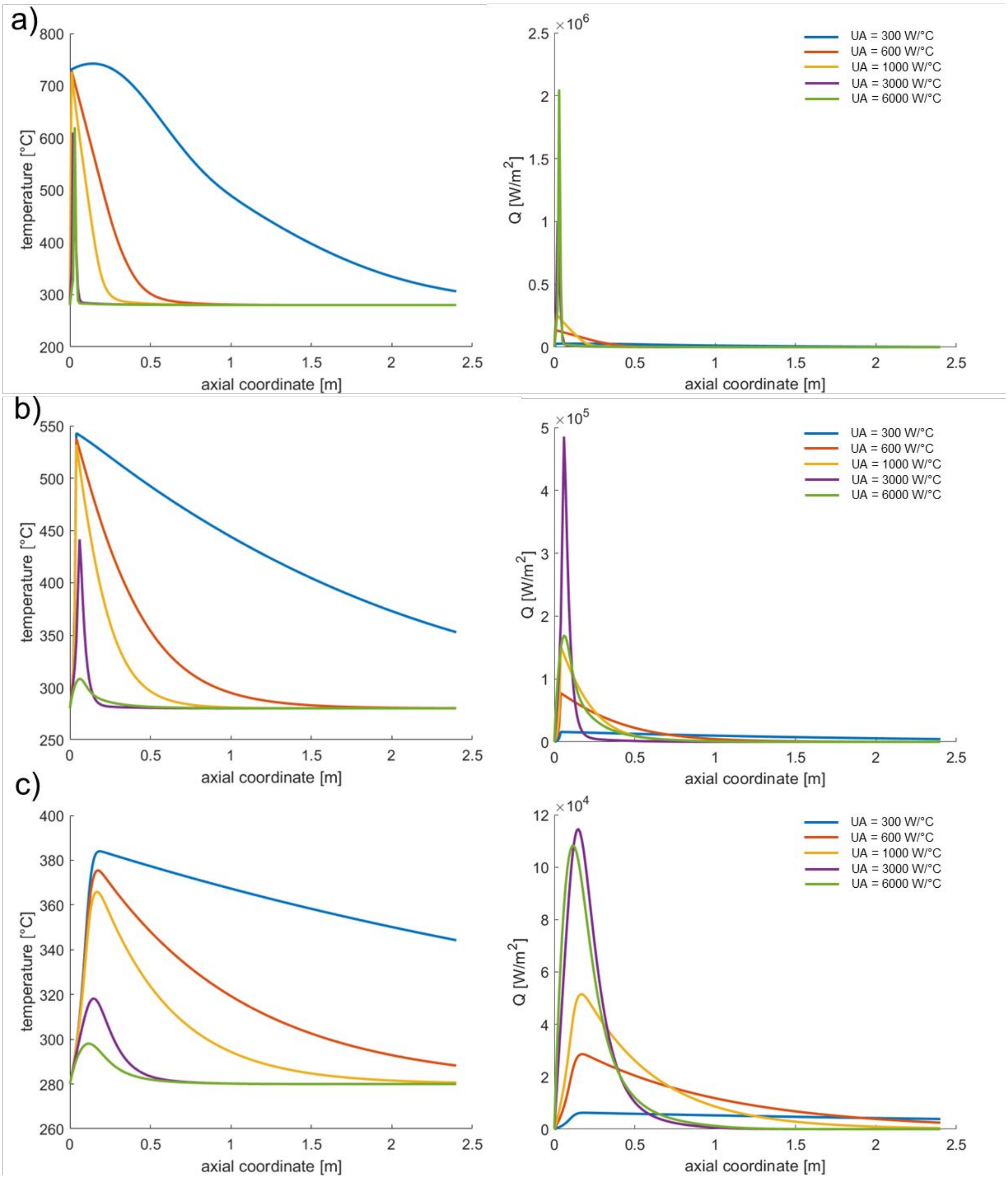

Figure S3. Simulation of biogas methanation reactor – Temperature [°C] (left) and heat transfer rate [W/m^2] (right) vs axial coordinate [m] for for Ni/Al_2O_3 diluted with SiO_2 (a), Al_2O_3 (b), SiC (c) varying the UA_{sez} values (colors). Experiment conditions: $CO_2/CH_4/H_2$=1/1.5/4, total flow rate equal to 5 Nm^3/h, p=8 bar, T=T_e=280°C, Φ=0.3.

[1] Moioli, E.; Senn, P.; Østrup, S.; Hütter, C. Results from the operation of an efficient and flexible large-scale biogas methanation system. *Energy Advances* **2024**, *3* (1), 131-142. https://doi.org/10.1039/D3YA00436H
